# Real-World Database Examining the Association between Sjögren’s Syndrome and Chronic Rhinosinusitis

**DOI:** 10.3390/jcm8020155

**Published:** 2019-01-30

**Authors:** Geng-He Chang, Yu-Cheng Chen, Ko-Ming Lin, Yao-Hsu Yang, Chia-Yen Liu, Meng-Hung Lin, Ching-Yuan Wu, Cheng-Ming Hsu, Ming-Shao Tsai

**Affiliations:** 1 Department of Otolaryngology – Head and Neck Surgery, Chiayi Chang Gung Memorial Hospital, Chiayi 613, Taiwan; genghechang@gmail.com (G.-H.C.); scm00031@gmail.com (C.-M.H.); 2 Department of General Medicine, Chiayi Chang Gung Memorial Hospital, Chiayi 613, Taiwan; u9702036@cmu.edu.tw; 3 Department of Rheumatology, Chiayi Chang Gung Memorial Hospital, Chiayi 613, Taiwan; koming@cgmh.org.tw; 4 College of Medicine, Chang Gung University, Taoyuan 333, Taiwan; 5 Health Information and Epidemiology Laboratory of Chang Gung Memorial Hospital, Chiayi 613, Taiwan; r95841012@ntu.edu.tw (Y.-H.Y.); qchiayen@gmail.com (C.-Y.L.); mattlin@cgmh.org.tw (M.-H.L.); 6 Department of Traditional Chinese Medicine, Chiayi Chang Gung Memorial Hospital, Chiayi 613, Taiwan; smbepigwu77@gmail.com; 7 School of Traditional Chinese Medicine, College of Medicine, Chang Gung University, Taoyuan 333, Taiwan

**Keywords:** Sjögren’s syndrome, sicca, autoimmune diseases, sinusitis

## Abstract

Objective: To investigate the risk of chronic rhinosinusitis (CRS) among patients with Sjögren’s syndrome (SS). Method: A total of 18,723 patients diagnosed with SS between 1997 and 2011 were retrospectively analyzed. Moreover, 59,568 patients without SS were matched to patients with SS at a 1:4 ratio on the basis of sex, age, urbanization level, income level, and the comorbidities of rhinitis and nasal sepal deviation. Patients were followed up until death or the end of the study period (31 December, 2013). The primary outcome was the occurrence of CRS. Results: The cumulative incidence of CRS was significantly higher in patients with SS than in those without SS (*p* < 0.001). The adjusted Cox proportional hazard model showed that patients with SS had a significantly higher incidence of CRS (hazard ratio, 2.51; 95% confidence interval, 2.22–2.84; *p* < 0.001). Sensitivity and subgroup analyses demonstrated SS was an independent risk factor for CRS. The dosage of intranasal corticosteroid spray used was not different between the SS and non-SS groups. Fewer patients with CRS in the SS group underwent sinus surgery (82/407 (20.2%)) than those in the non-SS group (179/667 (26.8%)) and this finding was statistically significant (*p* = 0.013). The number of operations did not differ significantly between patients with CRS in the SS and non-SS groups. Conclusions: SS is an independent risk factor for CRS. Our study extends the disease spectrum and prompts physicians to be aware of potential CRS occurrence after SS.

## 1. Introduction

Chronic rhinosinusitis (CRS), one of the most common diseases globally, continues to have a negative impact on patients’ quality of life [1]. Even with improvements in medications and surgical techniques in the past few decades, CRS still has a high prevalence of 2–16% worldwide [2]. A patient with CRS is estimated to lose approximately $200 U.S. dollars for every missed working day, and CRS results in an enormous health burden in many countries [2].

Sjögren’s syndrome (SS) is a chronic autoimmune disorder characterized by the lymphocytic infiltration of exocrine glands, particularly the salivary and lacrimal glands, which can lead to xerostomia and keratoconjunctivitis sicca [3,4,5]. In addition, SS may involve dryness and chronic inflammation of the sinonasal mucosa, which may cause CRS [3].

Min et al. reported systemic lupus erythematosus and rheumatoid arthritis as risk factors for CRS [6]. Lester et al. found a strong association between sicca symptoms and CRS [3]. SS is a major autoimmune disease, and patients with SS present typical sicca symptoms. Midilli et al. reported that among 77 patients with SS, two patients had sinusitis and among 77 healthy controls, one patient had sinusitis, without a statistically significant difference in either comparison [7]. Previous studies concerning the relationship between SS and CRS are scant, controversial, and lack long-term follow-up. To the best of our knowledge, the risk of CRS among patients with SS remains unknown. Therefore, in the present study, we investigated the effect of SS on the occurrence of CRS.

## 2. Materials and Methods

### 2.1. National Health Insurance Research Database

In March 1995, the Taiwanese government started the National Health Insurance Program, which currently covers 99.6% of Taiwan’s population [8]. The National Health Insurance Research Database (NHIRD) is a de-identified database that contains all medical claims that are data generated during reimbursement for insurance in an electronic format, including disease diagnoses for clinic visits and hospitalization, prescription drugs and doses, examinations, procedures, surgeries, payments, residential locations, and income levels for all beneficiaries. The diagnosis sets consist of three codes for outpatient visits and five codes for hospitalization. These codes are based on the International Classification of Diseases, Ninth Revision, Clinical Modification (ICD-9-CM).

We conducted this study in accordance with the guidelines of the Declaration of Helsinki. This study was granted exemption from the need for obtaining informed consent from participants, because the data were de-identified. All information of insurants was safeguarded to ensure their anonymity, and this study did not violate their rights or adversely affect their welfare. This study was approved by the Institutional Review Board (IRB) of Chang Gung Memorial Hospital (IRB number 201701329B1). The datasets generated and/or analyzed during the current study are available in the Taiwan National Health Insurance Research Database repository.

### 2.2. Study Group: Registry for Catastrophic Illness Patients

In the Taiwan National Health Insurance system, the Registry for Catastrophic Illness Patients (RFCIP) was established to enable a particular group of patients to benefit from the treatment of relevant catastrophic illnesses. Based on the disease definition in the registry system established using NHIRD, SS is categorized as a catastrophic illness, and patients with SS can apply for a catastrophic illness certificate. A patient with catastrophic illness certification is eligible for a large medical expense discount. The certification process requires critical evaluation of patients’ medical records, as well as serological and/or pathological reports by experts who are specialists in the disease field. Therefore, the diagnosis of enrolled patients with SS is highly accurate and reliable.

**Figure 1:**
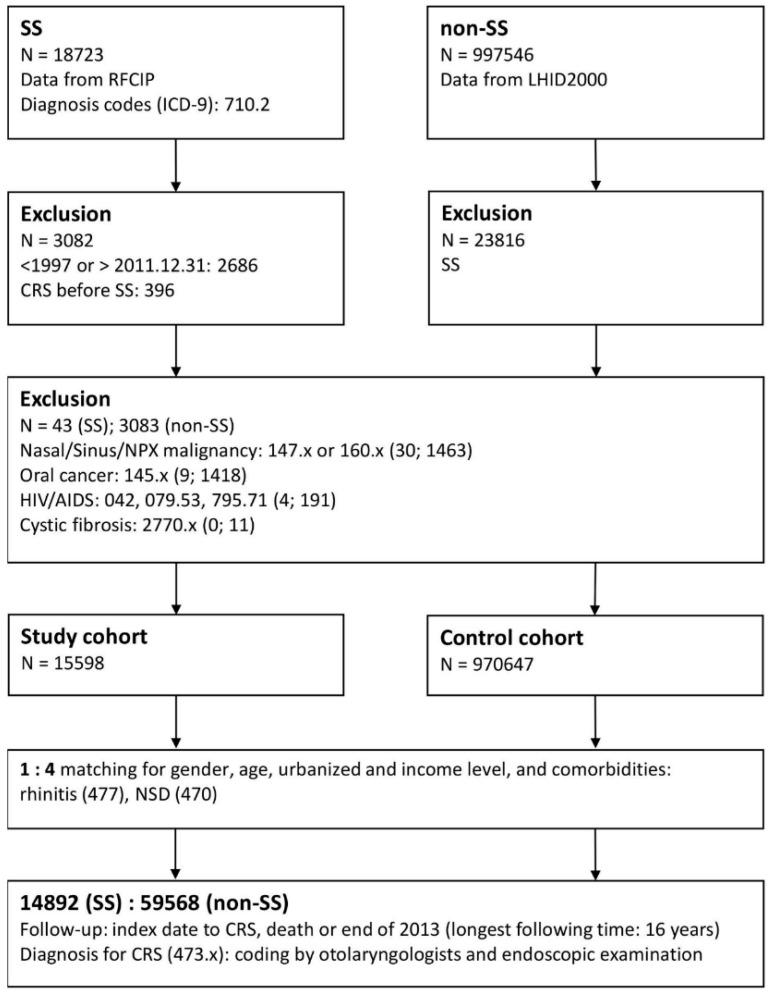
Enrollment flowchart of the study patients. Abbreviations: SS, Sjögren’s syndrome; RFCIP, Registry for Catastrophic Illness Patients; LHID2000, Longitudinal Health Insurance Database 2000; CRS, chronic rhinosinusitis; NPX, nasopharynx; HIV, human immunodeficiency virus; AIDS, acquired immune deficiency syndrome; NSD, nasal septal deviation.

From the RFCIP, we identified all patients diagnosed with SS (ICD-9-CM code 710.2) between January 1997 and December 2011 as our study group. Patients diagnosed with SS before 1997 and those diagnosed with SS after 2012 were excluded to confirm that SS was newly diagnosed and to ensure a follow-up period of at least 2 years. Patients were followed up until death or the end of the study period (31 December, 2013). We excluded patients with a previous diagnosis of CRS before SS to ensure the validity of our results concerning SS as a predisposing factor for CRS. We also excluded patients with malignancies such as nasal/sinus/nasopharyngeal cancer (ICD-9-CM code 147 × or 160 ×), oral cancer (ICD-9-CM code 145 ×), acquired immunodeficiency syndrome (AIDS; ICD-9-CM codes 042, 079.53, and 795.71), and cystic fibrosis (ICD-9-CM code 2770 ×). Those diseases were thought to be triggers for CRS, which would confound the results of the study. After exclusion, 15,598 individuals with SS were enrolled into the study group (Figure 1). The date of enrolment was defined as the date of the initial diagnosis date of SS.

### 2.3. Comparison Group: LHID2000 Database

The Longitudinal Health Insurance Database 2000 (LHID2000) consists of the data of 1 million patients randomly selected from NHIRD in 2000. According to a report by the National Health Research Institutes, no statistically significant differences exist in age, sex, or healthcare costs between the LHID2000 sample group and all enrollees in the NHIRD, and the LHID2000 has been used in several population-based studies [9,10,11]. Therefore, we used the LHID2000 to enroll patients without SS into the comparison group. According to the same exclusion criteria for the SS group, individuals with nasal/sinus/nasopharyngeal cancer, oral cancer, AIDS, and cystic fibrosis were excluded on the basis of the defined ICD-9-CM codes. For the comparison group, the start of the follow-up was defined as the date of the first visit to a medical facility in the year of enrolment. Patients diagnosed with CRS before enrolment were excluded from this study.

### 2.4. Matching Process

For comparison purposes, four patients without SS from the LHID2000 were matched to one patient with SS by sex, age, urbanization level, income level, and comorbidities. Comorbidities were defined as rhinitis (ICD-9-CM code 477) and nasal septal deviation (NSD; ICD-9-CM code 470). After the matching process, the sample comprised 14,892 patients with SS and 59,568 patients without SS in the study and comparison groups, respectively.

### 2.5. Main Outcome: Incidence of CRS

The primary outcome was the occurrence of CRS (ICD-9-CM code 473), which is defined as a diagnosis of CRS in inpatient settings, and in outpatient settings, the CRS diagnosis should fulfil the two following conditions: certified by an otolaryngologist and one or more than one endoscopic examinations received [12].

### 2.6. Comorbidities

The comorbidities associated with CRS were retrieved from the claims data: rhinitis (ICD-9-CM code 477) [13,14], NSD (ICD-9-CM code 470), gastroesophageal reflux disease (GERD) (ICD-9-CM codes 530.11 and 530.81) [15], chronic obstructive pulmonary disease (COPD) (ICD-9-CM codes 491, 492, and 496), asthma (ICD-9-CM code 493) [16], diabetes mellitus (DM) (ICD-9-CM code 250), hypertension (HT) (ICD-9-CM codes 401–405) [17], systemic lupus erythematosus (SLE) (ICD-9-CM code 710.0) [9], and rheumatoid arthritis (RA) (ICD-9-CM code 714) [18]. The comorbidities were included if they occurred one or more times in inpatient records or three or more times in outpatient records. Comorbidities were included if they occurred from 12 months before enrolment to the end of the follow-up period.

### 2.7. Analysis of CRS therapies

We analyzed the associated treatment for CRS in the SS and comparison groups, including the dosage of intranasal corticosteroid spray (INCS) used and the surgical intervention. Sinus surgeries included endoscopic and open procedures such as sinusectomy, sinusotomy, polypectomy, and Caldwell Luc operation.

### 2.8. Statistical Analysis

The demographic characteristics and comorbidities of the SS and non-SS groups were compared using a Pearson’s chi-squared test for categorical variables and an unpaired Student’s *t* test for continuous variables. Variables examined in univariate analysis that displayed a *p* value less than 0.1 were included in multivariate analysis. Kaplan–Meier analysis was used to estimate the cumulative incidence of CRS, and the differences between two groups were determined using a two-tailed log-rank test. Multivariable Cox proportional hazard regression models were applied to measure the hazard ratio (HR) and 95% confidence interval (CI) of CRS incidence in patients with SS in comparison with those of patients without SS. In addition, the stability of the effect of SS on CRS was examined through sensitivity and subgroup analyses. All analyses were performed using SAS software version 9.4 (SAS Institute, Cary, NC, USA), and the level of statistical significance was set at *p* < 0.05.

## 3. Results

Table 1 displays the baseline characteristics and comorbidities of the study and comparison groups. The SS group had a significantly higher prevalence of GERD, COPD, asthma, DM, SLE, and RA, but not of HT, than the non-SS group (Table 1). Among 14,892 patients with SS, 407 patients with CRS were identified in a mean follow-up period of 4.98 ± 3.62 years. By contrast, among 59,568 patients without SS, 667 patients with CRS were identified in a mean observation period of 5.2 ± 3.53 years. The incidence of CRS was significantly higher in the SS group than in the comparison group (*p* < 0.001).

**Figure 2:**
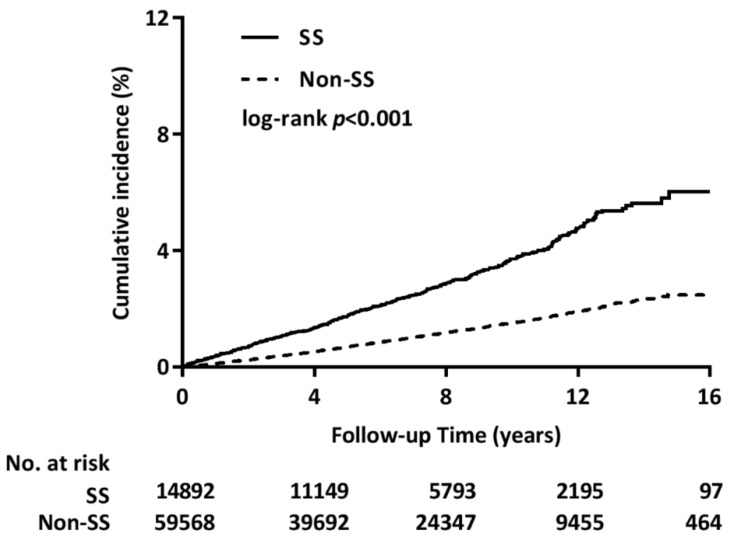
Cumulative incidence of CRS in patients with and without SS. SS, Sjögren’s syndrome. Patients with SS had a significantly higher cumulative incidence of chronic rhinosinusitis than those without SS.

The Kaplan–Meier method was used to estimate the cumulative incidence of CRS in both groups. The results indicated that the SS group had a significantly higher incidence of CRS than the non-SS group (*p* < 0.001) (Figure 2). The Cox proportional hazard model was used to estimate crude and adjusted HRs for both groups after adjustment for sex, age, urbanization level, income level, and the selected comorbidities, which were found to be significantly different in univariate analysis. The risk of CRS in patients with SS was 2.51-fold higher than that in patients without SS (adjusted HR, 2.51; 95% CI, 2.22–2.84; *p* < 0.001) (main model in Table 2). In addition, sensitivity analysis revealed a considerable stabilizing effect of SS on CRS occurrence. Subgroup analysis revealed that the effect of SS remained significant in the subgroup analysis for GERD, COPD, asthma, DM, HT, and RA.

Table 3 shows the associated therapies for CRS in the two groups. The dosage of INCS used was not significantly different between the SS and non-SS groups, regardless of whether vials or doses were counted. More patients received sinus surgery in the non-SS group than in the SS group (SS vs. non-SS, 82/407 (20.2%) vs. 179/667 (26.84%); *p* = 0.013). No difference was observed in the number of surgical interventions between the two groups (number of surgeries received per patient, SS vs. non-SS, 1.07 ± 0.19 vs. 1.14 ± 0.31; *p* = 0.064).

## 4. Discussion

Researchers have provided controversial conclusions regarding the predisposition of patients with SS toward CRS, and studies evaluating the relationship between SS and CRS are scant [3,7]. To the best of our knowledge, this is the first population-based study with long-term follow-up to investigate the risk of CRS in patients with SS. This study indicated that SS is a definite risk factor for sinusitis development, with a 2.5-fold risk.

SS is a systemic autoimmune disease and can thus affect exocrine glands, particularly the lacrimal and salivary glands, which can lead to the poor clearance of chronic inflammatory infiltrates [3]. Researchers have reported that SS can increase the risks of inflammation and infection in the lower respiratory tract. Nearly one-fourth of patients with SS have interstitial lung disease, and they also have potential risks of interstitial pneumonia, pleural disease, and community-acquired pneumonia [19,20,21,22]. However, SS might involve not only the lower airway but also the upper aerodigestive tract. Decreased secretion in the sinonasal tract may cause mucociliary function impairment, which may cause a thicker mucus layer with higher viscosity to be secreted and prolong mucociliary transport time [23]. Furthermore, SS may decrease the innate immune content concentration in the exocrine glands [24]. Decreased amylase and carbonic anhydrase in the secretion might impair innate immunity, which functions as a natural barrier [25,26]. SS also affects Meibomian glands, resulting in the increased evaporation of tears [27,28]. Without the moisture of natural tears from the nasolacrimal duct, the sinonasal cavity becomes drier, thus increasing the viscosity of mucus [29]. SS can lead to more frequent local inflammation and infection in the upper airway, as well as to small airway disease due to poor sputum clearance in the lower respiratory tract [30,31,32]. The aforementioned complications may explain our finding that SS is as a risk factor for CRS.

For CRS, saline nasal irrigation and INCS may be recommended as mainstay therapies. If medical treatment fails, surgical intervention should be considered [33,34,35]. In our study, SS patients with CRS did not use a higher amount of INCS than non-SS patients with CRS however, more patients received sinus surgery in the non-SS group than in the SS group, although the number of operations received per patient was not significantly higher. Because patients having CRS without nasal polyposis (CRSsNP) present with facial pain, pressure, and fullness more often than those having CRS with nasal polyposis (CRSwNP), which is frequently characterized by hyposmia and obstruction signs [36,37], patients with CRSwNP are more willing to accept sinus surgery to solve the annoying stuffiness affecting their breathing [37,38,39,40]. In Western countries, nearly 80% of individuals with CRSwNP are characterized by T-helper type 2 cell (Th2) cytokine expression, high tissue eosinophilia, frequent epithelial damage, a thickened basement membrane, and mostly edematous to sometimes fibrotic stromal tissue [41,42,43], which was thought to represent an inflammatory aetiology. By contrast, T-helper type 1 cell (Th1) cytokine expression is elevated in patients with CRSsNP compared with those with CRSwNP, with less eosinophilic infiltration [41,43], and infection is considered to be the main aetiology. Lin et al. reported GERD as a risk factor for CRS, and in their study, individuals with GERD and CRS had a higher incidence of CRSsNP (CRSsNP vs. CRSwNP, 48.80 vs. 19.59 persons per 10,000 person-years) [12]. Furthermore, Wong et al. reported that gastroesophageal reflux may stimulate the vagus nerve to cause a vagally mediated neural reflux between the esophagus and paranasal sinuses [44]. Other studies have also reported that the vagally mediated laryngotracheal reflux may trigger overproduction of mucus in the sinonasal tract and lead to sinus infections [45,46]. Therefore, considering the aforementioned information, we hypothesized that SS would be an infection-dominant aetiology for CRS, and that CRS might tend to be CRSsNP, which explains fewer surgical interventions in the SS CRS group than in the non-SS CRS group in our study. Further prospective study is needed to confirm our hypothesis.

Our study has several strengths. First, it was conducted using the RFCIP, which is affiliated with the NHIRD, and provides data of patients with accurate and reliable diagnosis of SS. Second, confirmation of CRS depends not only on ICD-9 codes but also on endoscopic examination and diagnosis specifically by otolaryngologists in the claims data. Third, our study included a large number of patients and conducted long follow-up, allowing us to present a nationwide overview.

Our study has some limitations. Patients with SS were identified using ICD codes instead of case collection. Therefore, in this study, information on clinical presentation and therapeutic course, laboratory data, radiological findings, and surgical and pathology reports were lacking, which are needed to investigate SS disease severity of and types of sinusitis (CRSsNP or CRSwNP).

## 5. Conclusions

To the best of our knowledge, our study is the first to verify SS as a definite risk factor for CRS within a five year follow-up period on average, with a 2.5-fold risk. Our study extends the SS disease spectrum and reminds physicians to be aware of potential CRS occurrence in patients with SS. Further research should be conducted to explore the characteristics of SS–CRS, methods to prevent CRS development, and improve treatment.

## Figures and Tables

**Table 1:** Baseline characteristics of study patients.

Characteristics	SS		Non-SS		*p*-value *
	*n*	%	*n*	%	
**Total**	14,892		59,568		
Gender					1
Male	1551	10.4	6204	10.4	
Female	13341	89.6	53,364	89.6	
Age (years)					1
<65	11742	78.9	46,968	78.9	
≥65	3150	21.2	12,600	21.2	
Urbanized level					1
1 (City)	4692	31.5	18,768	31.5	
2	6571	44.1	26,284	44.1	
3	2140	14.4	8560	14.4	
4 (Village)	1489	10.0	5956	10.0	
Income (NTD, per month)					1
0	3375	22.7	13,500	22.7	
1–15,840	2096	14.1	8384	14.1	
15,841–25,000	6390	42.9	25,560	42.9	
≥25,001	3031	20.4	12,124	20.4	
Comorbidities					
Rhinitis	5639	37.9	22,561	37.9	1
NSD	136	0.9	544	0.9	1
GERD	4378	29.4	8193	13.6	<0.001
COPD	2877	19.3	7605	12.8	<0.001
Asthma	2230	15.0	7086	11.9	<0.001
DM	2411	16.2	11,787	19.8	<0.001
HT	5837	39.2	23,690	39.8	0.200
SLE	3156	21.2	183	0.3	<0.001
RA	4299	28.9	1618	2.7	<0.001
CRS					
Total	408	2.7	667	1.1	<0.001

Abbreviations: SS, Sjögren’s syndrome; NTD, New Taiwan dollar; NSD, nasal septal deviation; GERD, gastroesophageal reflux disease; COPD, chronic obstructive pulmonary disease; DM, diabetes mellitus; HT, hypertension; SLE, systemic lupus erythematosus; RA, rheumatoid arthritis; CRS, chronic rhinosinusitis; * Statistical significance was expressed by *p* value < 0.05.

**Table 2:** Multivariable cox proportional hazard model of association between chronic rhinosinusitis and Sjögren’s syndrome.

Variables	HR	95% CI	*p*-value
Main model *	2.51	(2.22–2.84)	<0.001
Additional covariates ^†^			
Main model+GERD	2.52	(2.22–2.86)	<0.001
Main model+COPD	2.51	(2.22–2.84)	<0.001
Main model+Asthma	2.51	(2.22–2.84)	<0.001
Main model+DM	2.48	(2.20–2.81)	<0.001
Main model+HT	2.50	(2.21–2.83)	<0.001
Main model+SLE	2.57	(2.25–2.94)	<0.001
Main model+RA	2.69	(2.36–3.08)	<0.001
Subgroup effects			
GERD			
Yes	2.21	(1.73–2.83)	<0.001
No	2.64	(2.28–3.05)	<0.001
COPD			
Yes	2.16	(1.66–2.82)	<0.001
No	2.60	(2.26–2.99)	<0.001
Asthma			
Yes	2.18	(1.63–2.90)	<0.001
No	2.58	(2.25–2.96)	<0.001
DM			
Yes	2.71	(1.99–3.68)	<0.001
No	2.47	(2.15–2.82)	<0.001
HT			
Yes	2.74	(2.25–3.33)	<0.001
No	2.35	(2.01–2.76)	<0.001
SLE			
Yes	4.71	(0.65–33.9)	0.124
No	2.56	(2.24–2.92)	<0.001
RA			
Yes	2.25	(1.38–3.68)	0.001
No	2.71	(2.36–3.11)	<0.001

Abbreviations: HR, hazard ratio; CI, confidence interval; Abbreviations: SS, Sjögren’s syndrome; NTD, New Taiwan dollar; NSD, nasal septal deviation; GERD, gastroesophageal reflux disease; COPD, chronic obstructive pulmonary disease; DM, diabetes mellitus; HT, hypertension; SLE, systemic lupus erythematosus; RA, rheumatoid arthritis; CRS, chronic rhinosinusitis. * Main model is stratified by sex, age, urbanization, income, rhinitis, and nasal septal deviation. ^†^ The models are adjusted for covariates in the main model, as well as each additional listed covariate.

**Table 3:** Analysis of Medical and Surgical Therapies for CRS in SS and Non-SS.

	SS-CRS	Non-SS-CRS	
Characteristic	*n* = 407	*n* = 667	*p*-value *
INCS (mean ± SD)			
vial, per year	1.42 ± 1.71	1.50 ± 3.39	0.712
mg, per year	11.43 ± 18.36	13.02 ± 34.87	0.491
Surgery			
Yes or No	82 (20.2%)	179 (26.84%)	0.013
times (mean ± SD)	1.07 ± 0.19	1.14 ± 0.31	0.064

* Abbreviations: CRS, chronic rhinosinusitis; SS, Sjögren’s syndrome; INCS, intranasal corticosteroid spray; SD, standard deviation.

## List of abbreviations

GERD	gastroesophageal reflux disease
CI	confidence interval
CIP	Catastrophic Illness Patients
COPD	chronic obstructive pulmonary disease
CRS	chronic rhinosinusitis
CRSsNP	chronic rhinosinusitis without nasal polyposis
CRSwNP	chronic rhinosinusitis with nasal polyposis
DM	diabetes mellitus
HT	hypertension
HR	hazard ratio
ICD-9-CM	International Classification of Diseases, Ninth Revision, Clinical Modification
LHID2000	Longitudinal Health Insurance Database 2000
NHIRD	National Health Insurance Research Database
NSD	nasal septal deviation
RA	rheumatic arthritis
RFCIP	Registry for Catastrophic Illness Patients
SLE	systemic lupus erythematosus
